# The Expression of *stlA* in *Photorhabdus luminescens* Is Controlled by Nutrient Limitation

**DOI:** 10.1371/journal.pone.0082152

**Published:** 2013-11-22

**Authors:** Lea Lango-Scholey, Alexander O. Brachmann, Helge B. Bode, David J. Clarke

**Affiliations:** 1 Department of Microbiology, University College Cork, Cork, Ireland; 2 Molecular Biotechnology, Institute for Molecular Biosciences, Goethe University, Frankfurt, Frankfurt, Germany; Niels Bohr Institute, Denmark

## Abstract

*Photorhabdus* is a genus of Gram-negative entomopathogenic bacteria that also maintain a mutualistic association with nematodes from the family *Heterorhabditis*. *Photorhabdus* has an extensive secondary metabolism that is required for the interaction between the bacteria and the nematode. A major component of this secondary metabolism is a stilbene molecule, called ST. The first step in ST biosynthesis is the non-oxidative deamination of phenylalanine resulting in the production of cinnamic acid. This reaction is catalyzed by phenylalanine-ammonium lyase, an enzyme encoded by the *stlA* gene. In this study we show, using a *stlA-gfp* transcriptional fusion, that the expression of *stlA* is regulated by nutrient limitation through a regulatory network that involves at least 3 regulators. We show that TyrR, a LysR-type transcriptional regulator that regulates gene expression in response to aromatic amino acids in *E. coli*, is absolutely required for *stlA* expression. We also show that *stlA* expression is modulated by σ^S^ and Lrp, regulators that are implicated in the regulation of the response to nutrient limitation in other bacteria. This work is the first that describes pathway-specific regulation of secondary metabolism in *Photorhabdus* and, therefore, our study provides an initial insight into the complex regulatory network that controls secondary metabolism, and therefore mutualism, in this model organism.

## Introduction

*Photorhabdus* are Gram-negative bacteria that are pathogenic to insects whilst also maintaining a mutualistic association with nematodes from the family *Heterorhabditis*. The bacteria are normally found in the gut of the infective juvenile (IJ) stage of the nematode (for a recent review see [1]). The IJ is a non-feeding, soil-dwelling stage of the nematode that can infect a wide range of insect larvae. Once inside the insect host, the IJ releases its bacterial symbiont into the hemolymph of the insect where *Photorhabdus* reproduce exponentially and, in doing so, convert the tissues and organs of the insect into bacterial biomass. The insect normally dies 2-3 days post-infection and insect death is concomitant with the bacteria entering the post-exponential phase of growth [2,3]. At this time the IJ is also completing a specific developmental programme (called recovery) that culminates in the formation of a self-fertile adult hermaphrodite. The adult nematode now begins to feed on the bacterial biomass and lays eggs that progress through several juvenile molts to form a new generation of primarily self-fertile adult hermaphrodites (although a few males may also be present) [4,5]. This vegetative cycle continues for 2-3 generations until conditions within the insect cadaver deteriorate such that the developing juvenile nematodes are stimulated to enter an alternative developmental pathway (similar to diapause in the model nematode *Caenorhabditis elegans*) to become an IJ. As the IJs develop each one is colonized by *Photorhabdus* in a complex transmission process that has only recently been described [6,7]. The colonized IJs then emerge from the insect cadaver and begin to look for new hosts to infect.

The nematodes have an obligate requirement to feed on *Photorhabdus* during development within the insect and *in vitro*. During the post-exponential stage of growth *Photorhabdus* have been shown to express an elaborate secondary metabolism that includes the production of antibiotics, pigments and bioluminescence [8-11]. We have previously shown that mutants in *P. luminescens* TTO1 that were defective in expressing some products of secondary metabolism were also defective in their ability to support the growth and development of the nematode [8]. The virulence of these mutants was unaffected suggesting that the secondary metabolism of *Photorhabdus* is required for the mutualistic association with the nematode. We have been studying the role of individual secondary metabolites produced by *Photorhabdus* in mutualism, including a stilbene antibiotic (ST). We have shown that mutants that were unable to produce ST were defective in their ability to support nematode growth and development [12]. Indeed we have shown that ST appears to act as a signal for the nematode that stimulates the recovery of the IJ to the adult hermaphrodite. Therefore, the production of ST during the post-exponential phase of growth may be important in order to coordinate nematode development with bacterial growth.

Stilbenes are an important class of bioactive metabolites that are normally associated with plants. Resveratrol, for example, is a well-characterized stilbene with numerous documented beneficial pharmacological activities that is found in the skin of grapes [13-15]. Indeed, to our knowledge, *Photorhabdus* is still the only bacterium that has been shown to produce this class of molecule and recent genomic and prospective studies suggest that *Photorhabdus* may be an important source for the identification of novel bioactive molecules [16]. The first step in ST biosynthesis in *Photorhabdus* is the non-oxidative deamination of phenylalanine resulting in the formation of cinnamic acid (CA), a reaction that is catalyzed by the enzyme phenylalanine-ammonium lyase (PAL) encoded by the *stlA* gene [17]. The expression of *stlA* has been shown to increase during post-exponential growth although the regulators responsible for this induction were not identified [18]. Therefore, as part of our ongoing studies into the regulation of secondary metabolism in *Photorhabdus*, we wanted to identify factors involved in the regulation of *stlA*. In this way we were aiming get a better understanding of the regulatory networks involved in the control of secondary metabolism and mutualism in this bacterium.

## Materials and Methods

### Strains and plasmids

The wild-type TT01 strain used in all experiments was the spontaneous rifampicin-resistant mutant of *Photorhabdus luminescens* subsp. *laumondii* TT01 [19]. Luria-Bertani (LB) broth, Miller (Merck) (5g yeast extract, 10g peptone from casein and 10g sodium chloride per litre) was used for routine culturing. For solid media, 1.5% (w/v) agar (Merck) was added to LB broth. For culturing of *P. luminescens* on solid agar, LB was supplemented with 0.1% (w/v) pyruvate (according to [20]) and rifampicin. Unless stated otherwise, *P. luminescens* and *M. luteus* cultures were incubated at 30°C and *E. coli* cultures at 37°C, either statically or with agitation at 200 rpm. Antibiotics were added, when required, at the following concentrations: ampicillin (Amp) – 100 μg ml^-1^; chloramphenicol (Cm) – 20 μg ml^-1^; kanamycin (KnKn) – 30 μg ml^-1^; rifampicin (Rif) – 100 μg ml^-1^.

### Construction of stlA-gfp fusion

The *gfp* ^+^ gene from pZEP08 [21] is a variant of the *gfp* gene which carries the *gfp*_UV_ mutations F99S, M153T and V163A for enhanced brightness, along with the mutations F64L and S65T for more stable folding [22]. The *gfp*+ gene (including the ribosome-binding site) was amplified from pZEP08 using primers gfpF1 (*Kpn*I)(5’ - TTA TGG TAC CTT TAA GAA GGA GAT ATA CAT ATG - 3’) and fGFP_R2 (*Spe*I) (5’ - TTA TAC TAG TGC CAC CTG ACG TCT AAG AAA CC - 3’) and KOD DNA polymerase kit (Novagen). The amplicon was ligated into the mini-Tn7 vector, pEVS107 [23], resulting in the vector, pEVSgfp+. The entire mini-Tn7 region, as well as the part of the backbone containing the gene conferring KnKn resistance, was sequenced to confirm the integrity of the plasmid. The *stlA* promoter region (a 518bp region stretching from 485bp upstream of the start codon and including the first 11 codons of the *stlA* gene) was amplified from the TT01 genomic DNA using primers pStlA(*Kpn*I)F (5’ - TTA TGG TAC CCG ACC GTC ATA TTG CGA CCA ATC TG - 3’) and pStlA(*Kpn*I)R (5’ - TTA TGG TAC CGA AGA AAC GAA CTC CCT AAG GAT AC - 3’). The PCR product was digested with *Kpn*I and ligated into pEVSgfp+ digested with the same enzyme. In order to determine which colonies had the construct with the insert in the correct orientation, colony PCR was carried out using primers pStlA(*Kpn*I)F and fGFP_R2(*Spe*I). Colonies that produced the expected 1.5kb band were selected and the presence of the correct *stlA-gfp* fusion was confirmed by DNA sequencing. One plasmid was selected for further work and this new construct, pStlA-gfp+, was transformed into *E. coli* S17-1 λ*pir*. *P. luminescens* TT01, *E. coli* S17-1 λ*pir* p*StlA-gfp+* and E. coli S17-1 λ*pir* carrying the helper plasmid, pUX-BF13, were each inoculated into 30ml LB broth supplemented with 1mM MgCl_2_ (MgLB) and cultured, with agitation, until the OD_600_=0.5. The TT01 recipient cells, the *E. coli* S17-1 λ*pir* pUX-BF13 helper cells and the *E. coli* S17-1 λ*pir* p*StlA-gfp+* donor cells were then collected by centrifugation, washed and mixed together, in a total volume of 300μl, at a 4:1:1 ratio. The mixture was then transferred to the centre of an MgLB agar plate, allowed to dry, and the plate was incubated overnight at 30°C. The following day, the conjugation mix was plated out onto MgLB agar plates (supplemented with Km and Rif) and incubated at 30°C for 3 days. The resulting exconjugants were analysed by colony PCR to confirm the chromosomal insertion of the *stlA-gfp* fusion. As a control we also similarly constructed a derivative of TTO1 containing a chromosomal copy of a promoter-less *gfp*+ fusion ((-)-*gfp*) using pEVSgfp+.

### Construction of deletion mutants in *Photorhabdus*

The deletion mutants described in this study were constructed using a previously published protocol [24]. This method relies on homologous recombination to generate unmarked non-polar gene deletions, leaving only the start and the stop codons intact. DNA fragments (approx. 500bp) flanking the gene to be deleted are amplified using primer pairs (A+B and C+D). The 5’-end of primers B and C are compliementary and therefore, when mixed, the A+B and C+D amplicons form a chimeric molecule that can then be amplified with primers A and D. The full-length chimeric amplicon was cloned into a suicide vector, pDS132, encoding Cm^R^ and containing the *sacB* gene for counter-selection using sucrose [25]. The pDS132 clone was then conjugated into TTO1 using the donor strain *E. coli* S-17-1 λ*pir*. Exconjugants were selected by plating the conjugation mix on Rif and Cm and a number of single colonies were then cultured in LB broth (with no antibiotics) and serial dilutions were plated onto LB agar containing 0.2% (w/v) sucrose. Colonies with the correct resistance profile (Cm^S^ Suc^R^) were selected and the presence of the deletion mutation was confirmed by colony PCR and DNA sequencing. The plasmids used to delete *tyrR*, *rpoS*, *barA* and *uvrY* (pDS132-tyrR, pDS132-rpoS, pDS132-barA and pDS132-uvrY respectively) were constructed using primers described in Table S1. The pDS132-lrp plasmid was a gift from Prof Jon Clardy (Harvard University) [26]. The *rpoS* complementing plasmid, pBMM4292.2, was constructed by amplifying the *rpoS* gene and 800bp of the upsteam *nlpD* gene by PCR using the oligonucleotides: RJW190 (5’- TAATTAAGCTTCGTCAATGGGTGCGGCTGATAGC-3’) and RJW191 (5’- TATAAGCTTGCCAAACTATTCAACAATTAAGTACG-3’). The resulting amplicon was digested with *Hin*dIII and cloned into pBR322.

### Construction of the Tn7-complemented *ΔtyrR* mutant strain

A DNA fragment, containing the *tyrR* gene and promoter region (2,261bp in size) was amplified using primers F_tyrR-Tn7-com (5’ - TTA TCC TAG GCA GCA TTA ATG ATA GCA GTT AGC CCG - 3’) and R_tyrR-Tn7-com (5’ - TTA TAC TAG TTT ATT CGT CAC CAT CCG GCT CAT ATT CGG - 3’) to create a *Spe*I/*Avr*II-flanked DNA fragment. This amplicon was digested with *Spe*I and *Avr*II and was cloned into the mini-Tn7 vector pCIITn7K-a (a gift from Heidi Goodrich-Blair, University of Wisconsin-Madison). The integrity of the new plasmid, p*tyrR*-Tn7, was confirmed by DNA sequencing and the plasmid was transformed into the *E. coli* S17-1 λ*pir* donor strain for delivery into the TTO1 Δ*tyrR* mutant by triparental conjugation, as already described.

### Determination of fluorescence from bacterial cultures

For high-resolution fluorescence measurements using 96-well microtitre plates, strains containing the *stlA-gfp* or a promoter-less *gfp* ((-)-*gfp*) fusion were grown overnight in 5ml LB broth, supplemented with the appropriate antibiotics, and adjusted to an OD_600_ = 1. Black, optical bottom 96-well plates (Nunc) were filled with 145μl of fresh LB broth or modified M9 broth (with or without antibiotics) and 10μl of each adjusted overnight culture was added into the wells, in triplicate, and mixed well by pipetting. Modified M9 broth contains tryptone (10 g l^-1^) and yeast extract (5 g l^-1^) in a basal 1x M9 salts buffer (Na_2_HPO_4_ (6.66 g l^-1^), KH_2_PO_4_ (3 g l^-1^), NaCl (0.5 g l^-1^) supplemented with 2mM MgSO_4_. Each well was overlaid with 50μl of mineral oil before the plate was incubated inside a Synergy™ Multi-Mode Microplate reader (BioTek^®^), using the following settings: incubation at 30°C, absorbance and fluorescence readings every 15 min, shake before every reading for 15 s at the intensity level 2. The absorbance was read at 600nm, and the fluorescence was read from the bottom at the 485nm excitation and the 528nm emission wavelengths. The protocol was programmed using the KC4 software, and the results were exported into Excel for analysis. To calculate promoter activity the mean *stlA-gfp* fluorescence value at each time point (calculated from at least 3 biological replicates) was corrected for background by subtracting the fluorescence values measured from the (-)-*gfp* fusion at the same time point and in the same growth medium. The fluorescence data was then transformed into promoter activity using the equation; [ΔGFP/Δt]/OD_600_ [27]. In this equation ΔGFP represents the change (i.e. increase or decrease) in fluorescence over a change in time (Δt=0.25h in this study). This value is then normalized to cell biomass by dividing by the OD_600_ of the culture. 

For end-point assays cultures form 3 independent overnights for each strain (and the (-)-*gfp* control) were grown in 5 ml LB broth supplemented with the appropriate antibiotics. At regular intervals, 150μl from each sample were transferred into a clear, flat-bottom 96-well plate (Sterilin) for absorbance and fluorescence readings using a GENios XFLUOR4 plate reader (Tecan^®^). The absorbance was measured at 595nm; the fluorescence was measured at the 485nm excitation and the 535nm emission wavelengths. Three wells were filled for each individual sample, and the average fluorescence values were corrected for background before further analysis.

### Measuring ST production

For the overlay assay overnight cultures of *P. luminescens* TTO1 and the various mutants were grown in LB broth were diluted to an OD_600_=1 and 5μl was spotted onto the center of an LB agar plate. The plates were then incubated for 3 days at 30°C. In order to stop any further growth of *Photorhabdus* after this time, the colonies were exposed to UV light by removing the lids and irradiating the plates with UV light for 5 min. Each plate was then overlaid with soft LB agar (containing 0.75% (w/v) agar) that had been inoculated with *Micrococcus luteus*, a Gram-positive bacterium that is sensitive to the ST antibiotic. The plates were then incubated at 30°C for 3 days and the level of ST produced was determined by measuring the diameter of *M. luteus* growth inhibition surrounding the *Photorhabdus* colony. ST production was also measured using HPLC as previously described [12]. Therefore bacteria were cultured in LB broth with 2% Amberlite XAD-16 resin at 30°C. The ST was then eluted from the Amberlite using methanol and analysed using HPLC, as previously described [12].

### Whole cell fatty acid analysis

Whole-cell fatty acid analysis was carried out by GC-MS analysis of N-methyl-N-(trimethylsilyl) trifluoroacetamide (MSTFA) derivatized fatty acid methyl esters (FAME) as described elsewhere [28]. All analysis was performed with cell pellets from 1 ml bacteria culture adjusted to an OD_600_ of 11 after cultivation for 24 or 48 h at 30 °C and 200 rpm at a rotary shaker. Analysis was conducted on a 7890A model gas chromatograph (Agilent, Waldbronn, Germany) equipped with a CTC PAL Combi XT autosampler and coupled to a Series 5975C mass selective detector (Agilent, Waldbronn, Germany). For identification of the individual fatty acid methyl esters “Automated Mass Deconvolution and Identification Software” (AMDIS) version 2.64 was used with a fatty acid library.

## Results

### Expression of *stlA* during growth *in vitro*

**Figure 1:**
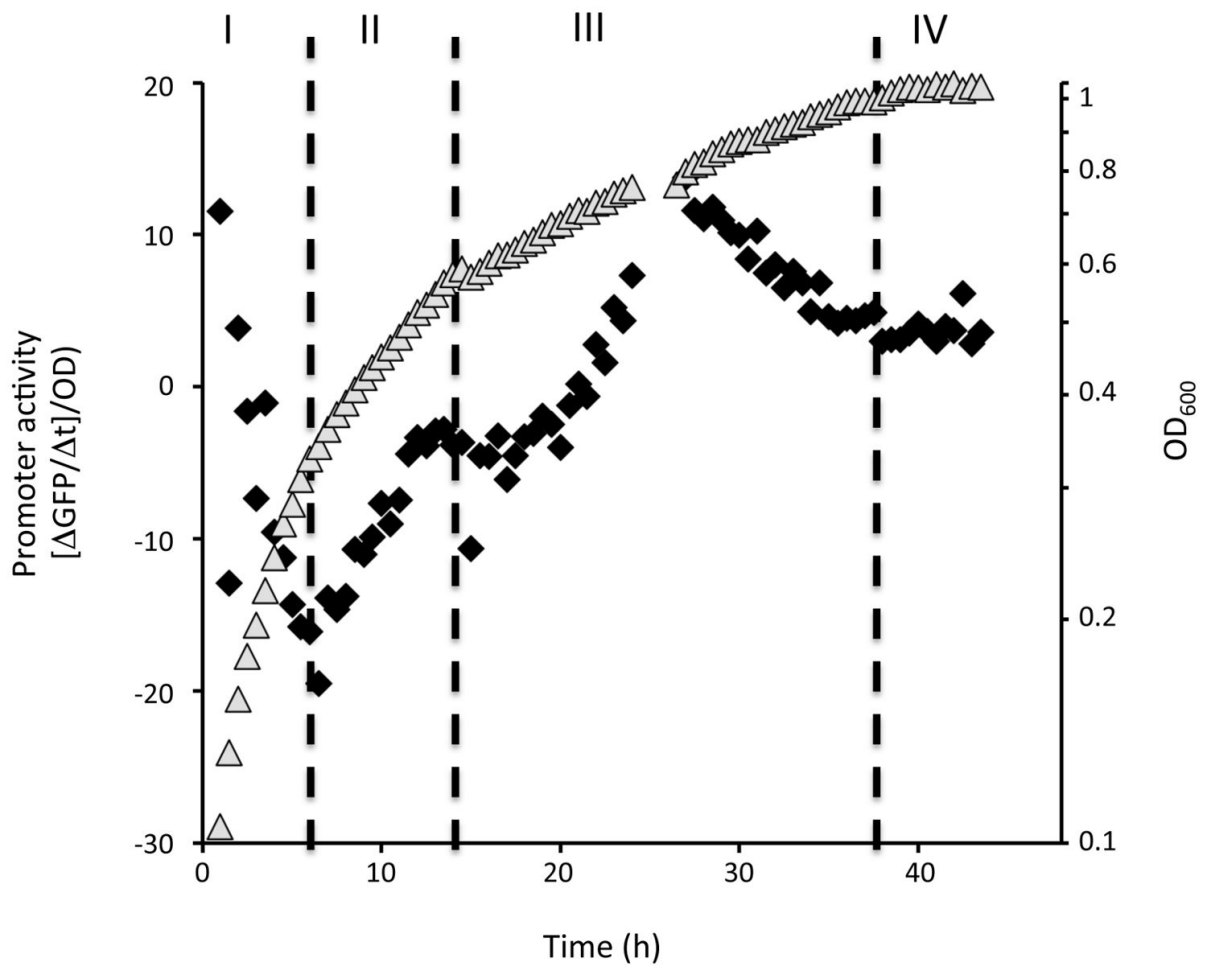
The activity of the *stlA* promoter during growth of *Photorhabdus* in LB broth. Cells carrying the *stlA-gfp* fusion were grown overnight and inoculated into the wells of a microtitre plate. The cells were cultured at 30°C and OD_600_ and fluorescence were measured at 15 min intervals for 43h. Promoter activity was calculated as described in Materials and Methods from at least 3 independent biological replicates. Promoter activity is represented by the filled diamond symbols whilst OD_600_ is shown as the filled grey triangles. Also shown are the 4 distinct growth phases (labeled I-IV) discussed in the text.

ST is an antibiotic that is one component of the extensive secondary metabolism produced by *Photorhabdus luminescens* TTO1 during post-exponential growth [3,11]. We have also shown that secondary metabolism, and ST, is required for symbiosis with the nematode partner [8,9]. Therefore, as part of our ongoing efforts to understand how secondary metabolism, and symbiosis, is regulated in *Photorhabdus*, we decided to look for regulators of ST production. The first step in the production of ST is the conversion of phenylalanine to cinnamic acid (CA) by the enzyme phenylalanine ammonia lyase, an activity encoded by the *stlA* gene [17]. We were interested in identifying transcriptional regulators of *stlA* expression and, to facilitate this, we constructed a single-copy, chromosomally-located *stlA-gfp* transcriptional fusion in *P. luminescens* TTO1 using Tn7. To characterize the expression profile of *stlA* during the growth of TTO1, cells carrying the fusion were grown in LB broth at 30°C and OD_600_ and fluorescence readings were taken every 30 min. Analysis of the OD_600_ suggests that there are 4 distinct growth phases (phase I-IV), defined by growth rate, exhibited by TTO1 during growth under these conditions (see Figure S1). The transitions between growth phases probably correspond to changes in the availability of nutrients as *Photorhabdus* has been shown to assimilate the amino acids present in LB broth in a specific order during growth [8]. Growth phase I is associated with rapid growth (0-6.5 h) and this is followed by 2 periods of slower growth rates (growth phase II=6.5-14 h and growth phase III=14-37.5 h) until growth phase IV (corresponding to stationary phase) is reached approximately 37.5 h post-inoculation (see Figure S1). In order to obtain a high-resolution visualization of *stlA* expression over time, the fluorescence readings gathered during this growth curve were converted into a measurement that represents *stlA* promoter activity (see Figure 1) [27]. Interestingly changes in the activity of the *stlA* promoter appear to correspond to the transitions through the different growth phases. Therefore the activity of the *stlA* promoter decreases during growth phase I before rising steadily during growth phase II. At this time the activity of the *stlA* promoter appears to remain steady for a period of 2-3 h before increasing rapidly again during growth phase III, reaching a maximum level of activity approximately 28h post-inoculation. The activity of the *stlA* promoter then begins to decrease again slowly until the end of growth phase III before leveling off as the cells enter growth phase IV (see Figure 1). As the different growth phases observed in this study probably correspond to changes in the assimilation of particular nutrients it would suggest that *stlA* expression is linked to the availability of nutrients (see Figure 1). Therefore *stlA* expression is temporally controlled during growth and may be regulated by nutrient availability.

### The expression of *stlA* is regulated by nutrient limitation

**Figure 2:**
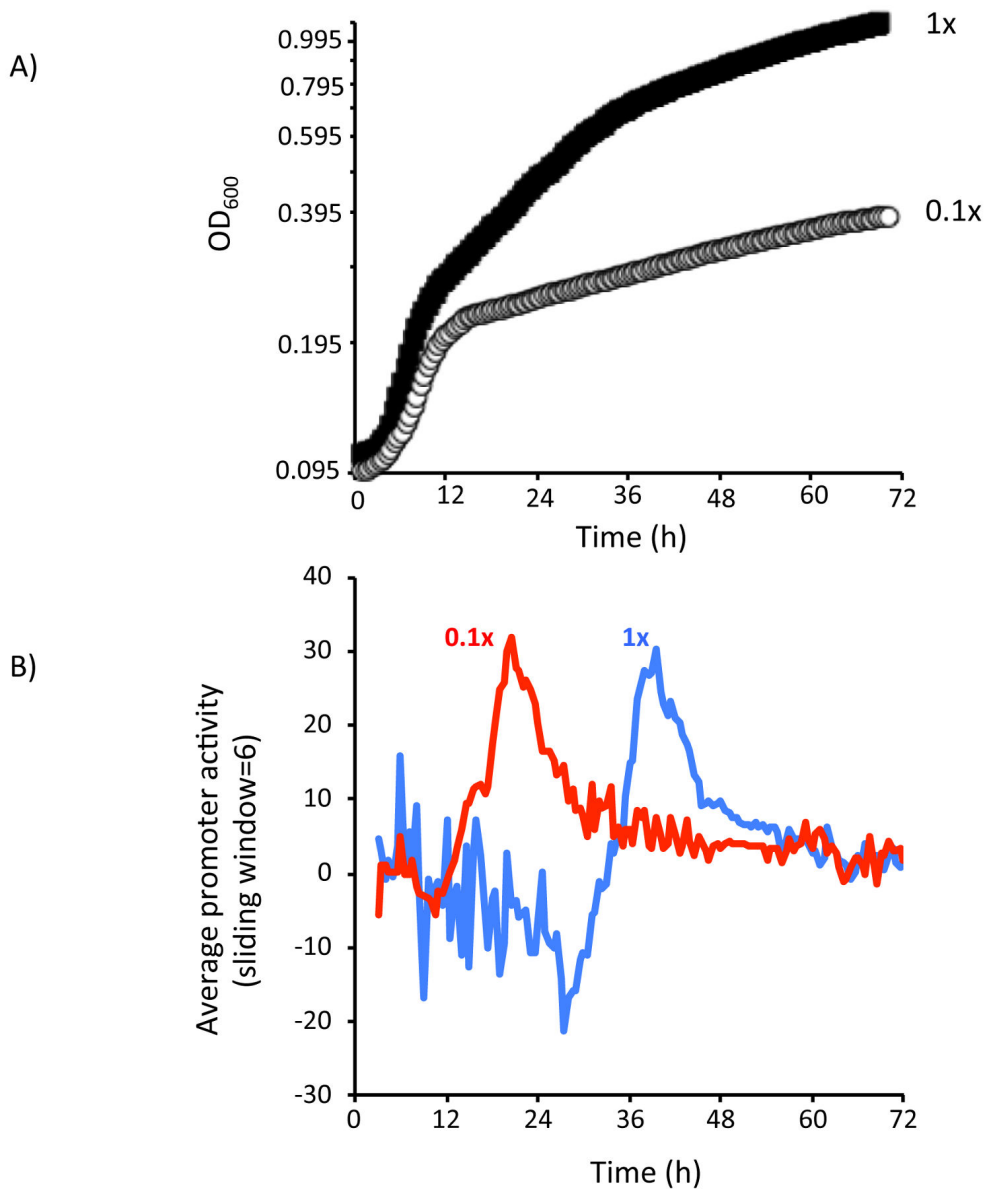
Nutrient limitation controls the activity of the *stlA* promoter. Cells carrying the *stlA-gfp* fusion were grown overnight and inoculated into the wells of a microtitre plate containing either 1x or 0.1x modified M9 broth, as indicated. The cells were cultured at 30°C and OD_600_ and fluorescence were measured at 15 min intervals for 72h. A) Growth curves of TTO1 containing the *stlA-gfp* fusion in 1x and 0.1x modified M9 broth. B) Promoter activity. The fluorescence associated with the *stlA-gfp* fusion in 1x and 0.1x modified M9 broth has been corrected for background by subtracting the fluorescence of the (-)-*gfp* control at each time point and grown in the same medium. The data was then transformed as described in Materials and Methods using the mean value of data from at least 3 independent biological replicates. In order to reduce noise (particularly at early time points) the activity of the *stlA* promoter during growth in 1x (blue trend-line) and 0.1x (red trend-line) is represented by a trend-line calculated using the average of a sliding window across 6 data points.

To confirm the role of nutrient availability in the regulation of *stlA* expression we decided to limit the nutrients in LB by diluting the only sources of carbon and nitrogen in this medium i.e. tryptone (TRY) and yeast extract (YE). To do this, we used a M9 salts based medium (called modified M9 broth) containing the same concentration of TRY and YE as LB broth (1% (w/v) and 0.5% (w/v), respectively). Growth of TTO1 in modified M9 broth appeared to be very similar to that observed in LB broth, including the different growth phases (compare Figure 1 and Figure 2). Moreover, similar to what we observed during growth in LB broth, *stlA* promoter activity is not seen to increase significantly until approx. 30 h post-inoculation during growth in 1x modified M9 broth. On the other hand, growth in M9 modified broth containing only 10% (w/v) of the TRY and YE (i.e. 0.1x M9 modified broth), slowed abruptly after approx. 12 h, implying that the cells were limited for carbon and/or nitrogen at this point. Interestingly this slow-down in growth coincided with an increase in *stlA* promoter activity (see Figure 2). Therefore *stlA-gfp* expression occurs at an earlier time-point, and at a lower cell density, in a nutrient-limited growth medium. This suggests that nutrient limitation plays an important role in the regulation of *stlA-gfp* expression. 

### TyrR is required for *stlA* expression and ST production

**Figure 3:**
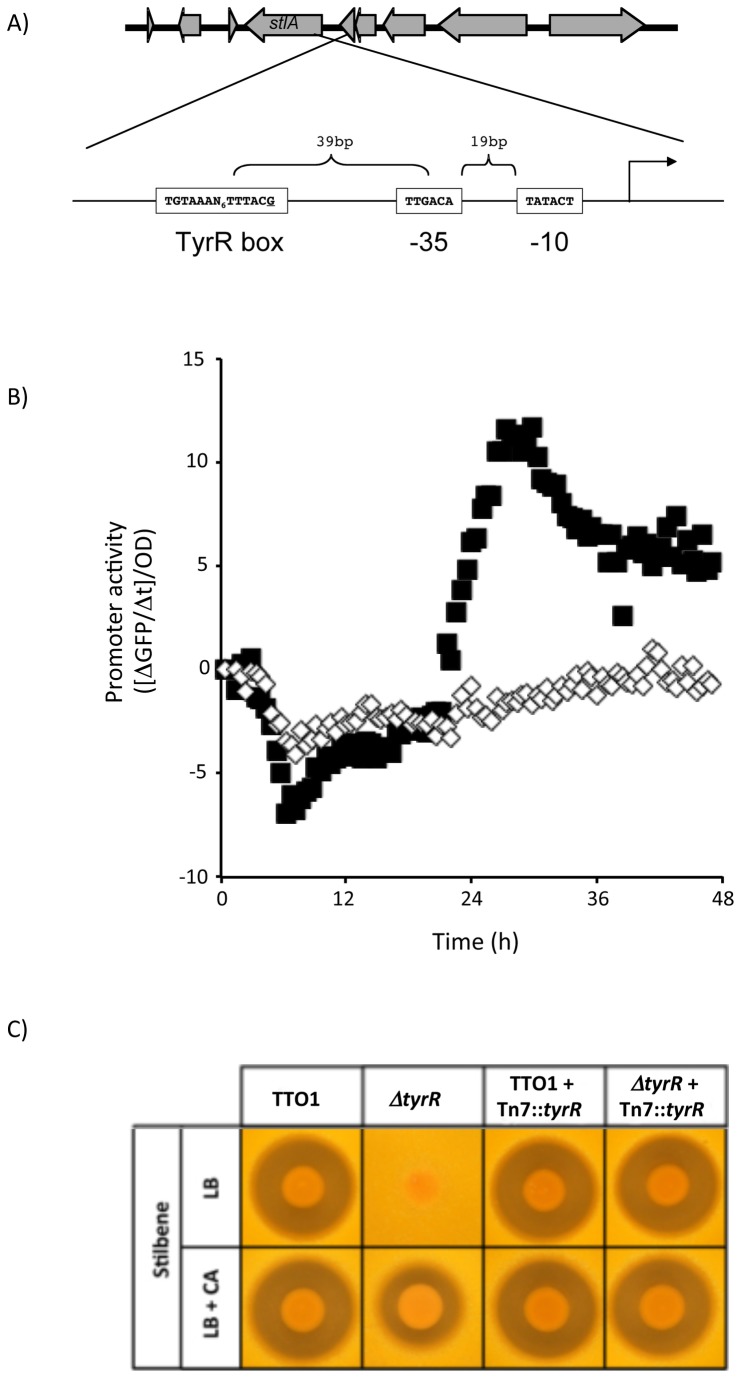
The *stlA* promoter requires TyrR for activity. A) The location of a predicted TyrR binding site upstream from the *stlA* promoter. The upper panel shows that location of the *stlA* gene on the TTO1 genome and the lower panel has been inverted with respect to the gene arrangement to facilitate the representation of the predicted TyrR binding site and promoter elements for the stlA gene. B) Promoter activity of the *stlA* gene in the Δ*tyrR* mutant. Wild-type (filled squares) or Δ*tyrR* mutant cells (open diamonds) carrying the *stlA-gfp* fusion were grown overnight and inoculated into LB broth in the wells of a microtitre plate. The cells were cultured at 30°C and OD_600_ and fluorescence were measured at 15 min intervals for 48h. Promoter activity was calculated as described in Materials and Methods using the mean of 6 independent biological replicates. C) TyrR is required for the production of ST. Cells, as indicated, were grown overnight and an aliquot was placed on LB agar, with or without 0.5mM cinnamic acid (CA), and incubated at 30°C for 3 days. At this time the colonies were overlaid with soft agar containing the ST-sensitive bacterium, *M. luteus*, and incubated for another 2-3 days at 30°C. Inhibition of growth of the *M. luteus* is indicative of ST production. Shown is a representative experiment from at least 3 biological replicates.

In a previous transposon mutagenesis study we identified several mutants in *P. luminescens* TT01 that were unable to produce the ST antibiotic, as determined by an overlay assay using *Micrococcus luteus* as the reporter strain [8,12,17]. One of the previously unpublished mutants from these studies was in a gene with homology to *tyrR*, encoding a LysR-type transcriptional regulator, from *Escherichia coli*. The TyrR regulon in *E. coli* has been sufficiently characterized to facilitate the identification of a TyrR-binding DNA consensus sequence, TGTAAA-N_6_-TTTACA [29]. Using this sequence as a search query we identified a near perfect (1 mismatch) TyrR-binding site centered 133bp upstream from the first base pair of the predicted start codon of *stlA* (see Figure 3A). The predicted TyrR binding site is also located 39 nucleotides upstream from a potential -35 element for the binding of σ^70^ (see Figure 3A). To confirm the involvement of TyrR in the expression of *stlA* we constructed a clean deletion mutant of *tyrR* (Δ*tyrR*) in the *stlA-gfp* background and measured promoter activity during growth in LB broth. There was very little activity from the *stlA* promoter at all stages during the growth of the Δ*tyrR* mutant confirming that *tyrR* encodes an important positive regulator of *stlA* expression (see Figure 3B). As expected the Δ*tyrR* mutant was unable to produce ST (as measured using the overlay assay) and this defect was rescued by supplementing the growth medium with CA (see Figure 3C). Finally ST production in the Δ*tyrR* mutant could be complemented by the *in trans* expression of an intact copy of the *tyrR* gene that was inserted into the genome using Tn7 (see Figure 3C). Therefore the expression of *stlA* requires the presence of the TyrR transcriptional regulator.

**Figure 4:**
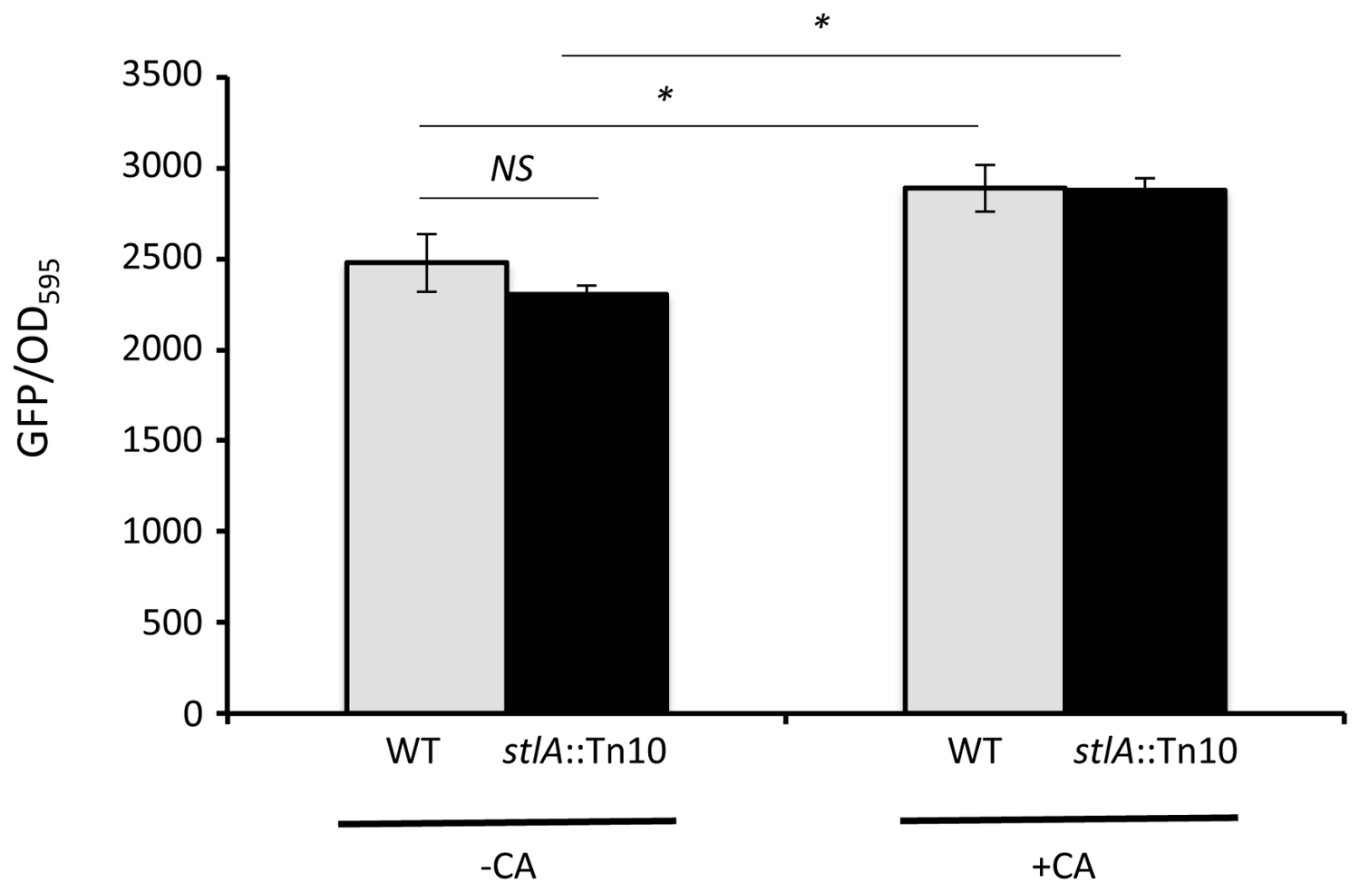
Cinnamic acid can positively regulate *stlA* expression. Wild-type (WT) or *stlA*::Tn10 mutant cells carrying the *stlA-gfp* fusion were grown for 72h at 30°C in LB broth in the presence or absence of 0.5mM cinnamic acid (as indicated). OD_595_ and fluorescence readings were taken and the results shown are the mean of at least 3 experiments and the error bars represent the standard deviation. Statistical significance was tested using the Student T-test and differences were deemed significant if *P*<0.05 (*). *NS*: not significant.

In *E. coli* the activity of TyrR is affected by the presence of aromatic amino acids, including phenylalanine (Phe) [29-31]. However we could not detect any significant affect on the activity of the *stlA* promoter following the addition of Phe to LB broth (data not shown). CA is derived from Phe and it was possible that this metabolite might affect the activity of TyrR and, thus, the expression of *stlA*. Although CA is not required for stlA expression we did see a small, but significant, increase in the level of *stlA* expression when TTO1 was cultured for 72h in the presence of 0.5mM CA in LB broth (see Figure 4). However the role of TyrR in this regulation, if any, is unclear. 

### Lrp and σ^S^ positively regulate *stlA* expression

**Figure 5:**
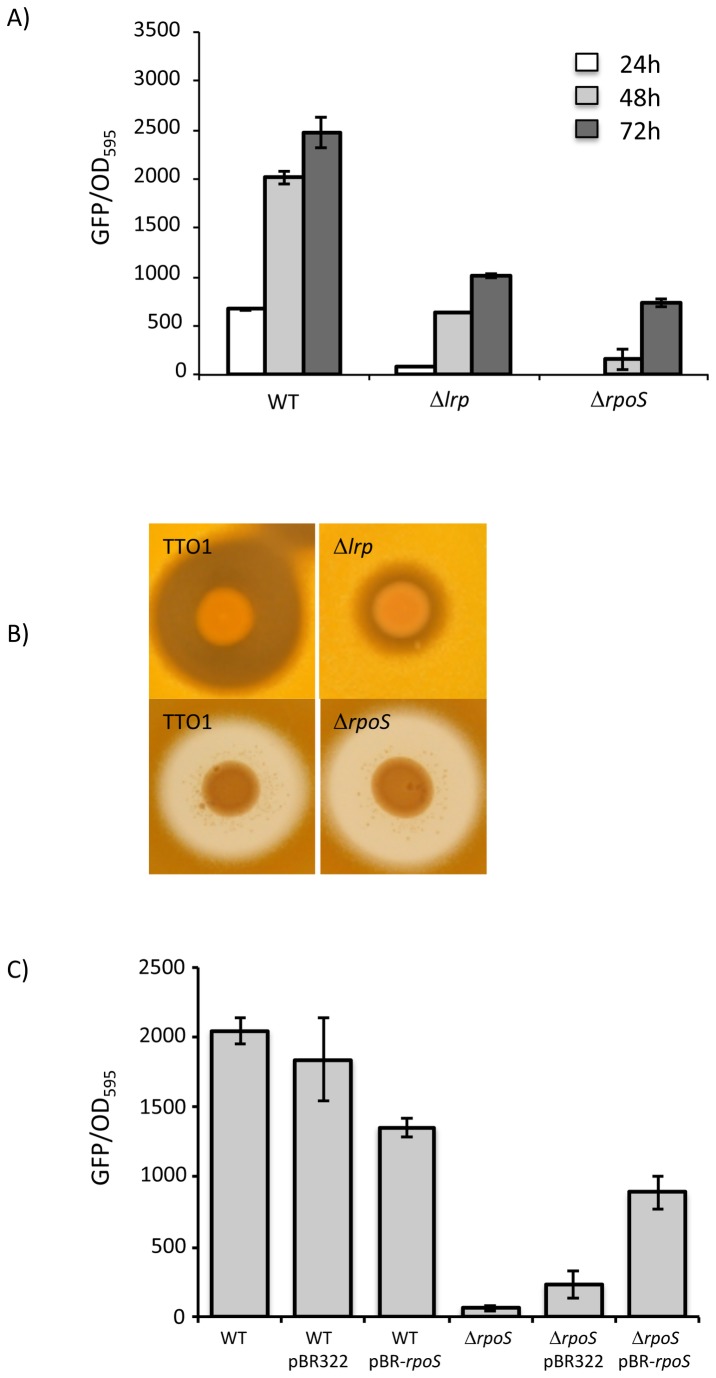
Both *lrp* and *rpoS* are required for normal *stlA* expression. A) The WT and mutant strains containing the *stlA-gfp* fusion were cultured in LB broth and, at the indicated times, samples were taken for OD_595_ and fluorescence readings. The results shown are the mean of at least 3 experiments and the error bars represent the standard deviation. B) The WT and mutant strains were aliquoted onto LB agar and cultured for 3 days at 30°C. Colonies were then overlaid with soft agar and the ST-mediated inhibition of the growth of *M. luteus* was observed after a further 2-3 days incubation at 30°C. The difference in appearance of the zones of inhibition between the upper and lower panels is due to different lighting conditions used to take the PHOTOs. C) Complementation of *stlA-gfp* expression in the Δ*rpoS* mutant. WT or Δ*rpoS* mutant cells were transformed with vector alone (pBR322) or with a copy of the *rpoS* gene under the control of its own promoter, pBMM4292.2 (pBR-rpoS). Cultures were grown in LB broth (supplemented with Amp where appropriate) and samples were taken for fluorescence and OD_595_ after 48h incubation at 30°C. Values shown are average of 3 independent experiments and the error bars represent standard deviation.

Although TyrR is required for *stlA* expression it is likely that other regulators are going to play a role in modulating the timing and level of expression of this gene. Both σ^S^ and Lrp are known to play an important role in the regulation of gene expression in response to nutrient limitation in closely related bacteria such as *E. coli* and *Xenorhabdus* [32-34]. Therefore we constructed clean deletion mutants of *rpoS* and *lrp* in TTO1 and measured the affect on *stlA* expression using the *stlA-gfp* fusion. During these experiments cells were grown in LB broth in flasks with agitation and samples were taken for OD_600_ and fluorescence readings at 24, 48 and 72 h post-inoculation. Both Δ*lrp* and Δ*rpoS* mutant strains show significantly reduced levels of *stlA-gfp* expression at all time points tested suggesting that these regulators are required for normal *stlA* expression (see Figure 5A). Interestingly, despite the decreased level of *stlA* expression in the Δ*rpoS* mutant (2-3 fold lower than WT), the level of ST production was normal (See Figure 5B). Therefore despite the reduction in *stlA* expression, and presumably the StlA activity, the level of CA produced by this mutant does not appear to be limiting for ST biosynthesis. Indeed, it has previously been reported that only a small fraction of the total CA produced by *P. luminescens* TTO1 is used for ST biosynthesis [18]. We were able to complement the defect in *stlA-gfp* expression in the Δ*rpoS* mutant by expressing an intact copy of *rpoS in trans* from a plasmid, pBMM4292.2 (see Figure 5C). The level of pBMM4292.2 retention in the Δ*rpoS* cells at the time of analysis (48h post-inoculation) was < 7% and this would explain the partial complementation observed.

On the other hand, although the decrease in expression of *stlA* in the Δ*lrp* mutant was equivalent to that observed in the Δ*rpoS* mutant, the Δ*lrp* mutant strain did not produce normal amounts of ST, as determined by both the overlay assay and by HPLC (see Figure 5B and Figure S2). Interestingly HPLC analysis also confirmed lower levels of CA present in the supernatants of the Δ*lrp* mutant (see Figure S2). We have previously shown that ST biosynthesis involves 2 converging branches, one branch originates in phenylalanine metabolism and the other branch originates in branched chain amino acid metabolism [12]. StlA catalyzes the first step in the phenylalanine-dependent branch whilst the other branch involves a branched-chain α-ketoacid dehydrogenase (Bkd, encoded by the *bkdABC* operon) that is required to convert isoleucine to isovalerate-CoA, a precursor of ST [12,17]. Isovalerate is also a precursor for the production of branched-chain fatty acids (BCFA) and we have previously shown that BCFA are a significant component of the fatty acids present in *P. luminescens* TTO1 [12]. Indeed the expression of *bkdABC* in *E. coli* has been shown to result in the production of BCFA in this bacterium [28]. Therefore it is possible that Lrp might be involved in the regulation of both *stlA* and *bkdABC*. In order to determine whether the expression of the *bkdABC* operon might be reduced in the Δ*lrp* mutant we decided to quantify the levels of BCFA in TTO1 and the Δ*lrp* mutant using FAME analysis. Cells were grown in LB broth for 24 and 48h and total fatty acids were extracted according to Materials and Methods. There was a clear decrease in the overall level of BCFA ((ante)isoC15:0, isoC16:0, isoC16:1, isoC17:0, (ante)isoC17:0 and isoC17:1) present in the Δ*lrp* mutant compared to the wild-type (38.5% total BCFA vs 50.3% total BCFA after 24h and 28.3% total BCFA vs 49.7% total BCFA after 48h, respectively) with a corresponding compensatory increase in the level of C16:0 and C12:0 in the Δ*lrp* mutant (see Table 1). Therefore we have shown that the expression of *stlA* and the activity of Bkd are reduced in the Δ*lrp* mutant and the combination of these phenotypes could explain the reduced production of ST observed in this mutant. 

**Table 1:** Total fatty acid analysis of TTO1 and the Δ*lrp* mutant.*

	Cells from 24 h cultures		Cells from 48 h cultures	
	Δ*lrp* [%]	TT01 [%]	Δ*lrp* [%]	TT01 [%]
12:0	6	--	7.4	--
14:0	--	3.6	--	3.5
16:0	25	18.5	30.2	18.2
16:1w7c	13.3	12.2	16	11.3
18:1	8.2	11.7	10.9	13
**iso15:0**	**22.3**	**20.4**	**14.9**	**17.8**
**anteiso15:0**	**2.6**	**3.5**	**1.5**	**3.2**
**iso16:0**	**0.9**	**1.8**	**0.9**	**1.8**
**iso17:1**	**3.4**	**5.9**	**2.9**	**6.5**
**iso17:0**	**7**	**14**	**5.9**	**15**
**anteiso17:0**	**1**	**3.7**	**0.8**	**4.4**
14:0 3-OH	2.7	1.5	5	2.5
**iso15:O 3-OH**	**1.3**	**1**	**1.4**	**1**
16:0 9,10CH_2_	4.6	0.9	1.3	0.7

* : Fatty acids present at levels <0.5% of total fatty acids were excluded.

--: not detected

Note: the branched-chain fatty acids (BCFA) are in bold font.

## Discussion

*Photorhabdus* has an extensive secondary metabolism that is required for the symbiotic relationship with their nematode partner, *Heterorhabditis* [8,9,11]. A major component of this secondary metabolism is ST, a molecule that, in addition to having antimicrobial activity, has an important role as an inter-kingdom signal that controls the development of the nematode partner [12]. The first step in the biosynthesis of ST requires phenylalanine ammonium-lyase (encoded by the *stlA* gene), an enzyme that deaminates the aromatic amino acid, phenylalanine resulting in the formation of CA [17]. In this study, we undertook to characterize the regulation of *stlA* expression as part of our on-going studies into the regulation of secondary metabolism in *Photorhabdus*.

It has previously been shown that the expression of *stlA* increased during the post-exponential phase of growth of *P. luminescens* TTO1 [18]. We have confirmed this observation here and we have further shown that *stlA* expression (and the activity of the *stlA* promoter) is regulated by the availability of nutrients. Therefore when 0.1x modified M9 broth is used as the growth medium the expression of *stlA* occurs earlier, and at a lower cell density, compared to cells grown in 1x modified M9 broth (see Figure 2). Amino acids and peptides are the major source of carbon and nitrogen in the modified M9 broth and this suggests that, under these conditions, amino acid limitation is triggering *stlA* expression. The role of nutrient limitation in the transition from primary (i.e. biomass production) to secondary metabolism has been well-studied in bacteria although the underlying mechanisms are only now being elucidated [35,36]. In actinomycetes (the model organisms used for the study of secondary metabolism) it has been shown that the depletion of nutrients triggers an alteration in metabolic flux (i.e. a metabolic switch) within the cell resulting in a transition away from the production of biomass (i.e. primary metabolism) towards the production of small bioactive compounds (i.e. secondary metabolism) [35,37]. This metabolic switch contributes to a regulatory network that links biomass production with the expression of the genes involved in secondary metabolism [35,38]. Similar metabolic switches have been identified in bacteria other than actinomycetes. We have previously identified a metabolic switch in *P. luminescens* and we have shown that the TCA cycle plays an important role in the regulation of secondary metabolism [8]. Therefore mutants in the TCA cycle (specifically deletion mutations in *mdh* or *fumC*) completely block secondary metabolism. Indeed there is very little *stlA-gfp* expression in the Δ*mdh* mutant (our unpublished data). However the regulatory network that connects the TCA cycle with secondary metabolism in *Photorhabdus* has not been characterized. In *Pseudomonas fluorescens* CHAO, a similar relationship between the TCA cycle and secondary metabolism has been reported although, in contrast to *P. luminescens*, mutations in *fumA* resulted in the up-regulation of secondary metabolism genes in this bacterium [39]. Subsequent work identified the GacS-GacA two-component pathway (also known as the BarA-UvrY pathway) in *P. fluorescens* CHAO as having an important regulatory role in this metabolic switch [39,40]. In *P. luminescens*, it has been shown that UvrY positively regulates the expression of a number of genes predicted to be involved in secondary metabolism, including *stlA* [41]. Therefore a lower level of *stlA* mRNA (x13-fold reduced) was detected in the *uvrY* mutant strain compared to the P. *luminescens* TTO1 wild-type strain at the end of exponential growth [41]. In the case of *stlA* this reduction in mRNA levels was shown to be due to the fact that the regulatory RNA, *csrB*, is not expressed in the *uvrY* mutant [41]. The *csrB* sRNA works by binding the RNA-binding protein, CsrA, thus preventing CsrA from binding its mRNA targets [42]. Therefore, in *P. luminescens* it would seem likely that, in the absence of *csrB* expression, the *stlA* transcript is destabilized due to the binding of CsrA. However, despite the significant reduction in *stlA* transcript levels, the level of ST produced in the *uvrY* mutant does not appear to be reduced compared to the wild-type (our unpublished data and [26]). As previously mentioned this can be explained by the fact that only a fraction of the CA produced is required for normal ST production [18]. Therefore the BarA-UvrY two-component pathway is, at best, only part of the regulatory network that interacts with the metabolic switch in *Photorhabdus*. 

The timing of the metabolic switch in *Photorhabdus* coincides with the transition from exponential to post-exponential growth and this is, undoubtedly, also linked to the availability of nutrients. In *E. coli* (and other bacteria) the alternative sigma factor, σ^S^, has been shown to be an important regulator of the transition from exponential to post-exponential growth [43]. In this study we have shown that σ^S^ does play a significant role in the expression of *stlA*. Although we have not established that σ^S^ directly binds to the *stlA* promoter we have identified a sequence, overlapping the proposed -10 box in the *stlA* promoter, that is nearly identical (8 out of 9 nucleotides) to the consensus sequence of the extended -10 box that has recently been shown to be indicative of σ^S^-controlled promoters in *E. coli* (see Figure S3) [1,4]. Nonetheless, whether regulation is direct or indirect, our data demonstrates that σ^S^ contributes to the regulation of *stlA* and is required for maximal CA production during post-exponential growth. 

Lrp is another transcriptional regulator that is involved in controlling the transcriptional response of cells to the nutritional status of the environment [44]. Lrp controls the expression of genes involved in amino acid metabolism and transport and the activity of Lrp can be modulated by the presence of particular amino acids e.g. leucine [45]. In this study, we have shown that Lrp is required for the normal expression of *stlA* in *Photorhabdus*. However it has not yet been established whether the affect of Lrp on the *stlA* promoter is direct or indirect. Interestingly, Lrp can affect σ^S^ promoter selectivity in *E. coli* and mutations in *lrp* have been shown to reduce the σ^S^-dependence of certain promoters [4]. Indeed approximately 50% of the σ^S^-regulated genes identified by microarray analysis have also been shown to be regulated by Lrp [4,44]. A recent genome-scale reconstruction of the Lrp regulatory network in *E. coli* identified a 15bp sequence motif that is structured with flanking CAG/CTG triplets and a central AT-rich signal [46]. We could not detect this motif in the *stlA* promoter region although Lrp binding site specificity can change in the presence or absence of co-factors such as leucine. In this study we have shown that, in addition to *stlA* expression, Lrp is also required for normal BCFA biosynthesis in *Photorhabdus* (see Table 1). BCFA production is very unusual in the *Enterobacteriaceae* and we believe that it is the reduction in BCFA production that is limiting the level of ST production observed in the Δ*lrp* mutant. Interestingly, and in contrast to our data, it has been reported that there is a modest increase in ST production in a Δ*lrp* mutant strain of *P. luminescens* TTO1 [26]. One possible explanation for this discrepancy might be the presence of lineage-specific genotypic differences between strains of *P. luminescens* used in different laboratories. For example, it is well established that different alleles of *rpoS* exist within the same strain of *E. coli* and these can be selected for during the *in vitro* culturing of these bacteria [47,48]. It is therefore possible that different lineages of *P. luminescens* TTO1 have emerged in different laboratories and the plasticity of the TTO1 genome has already been established in comparative studies [49]. Moreover, the use of NMR by Kontnick and colleagues to specifically measure the level of ST produced in their Δ*lrp* mutant may also contribute to this apparent discrepancy [26]. *Photorhabdus* is known to produce a number of molecules that are derived from ST and it is not clear what contribution (if any) these derivatives make to the overall antimicrobial activity observed in *Photorhabdus* [26,50]. Therefore, whilst the production of ST (specifically) may be elevated in the Δ*lrp* mutant produced by Kotnick and colleagues the overall profile of ST derivatives produced in this Δ*lrp* mutant might be different and this might result in a reduction in the antimicrobial activity of *Photorhabdus* when measured using a crude assay such as the overlay assay (although this was not tested).

In addition to showing that both σ^S^ and Lrp contribute to the regulation of *stlA* expression, we have also shown that a LysR-type transcriptional regulator, TyrR, is absolutely required for the expression of *stlA*. TyrR has been shown to regulate the expression of genes involved in aromatic amino acid metabolism and transport in a number of bacteria, including *E. coli*, *Shewanella* and *Erwinia* [31,51,52]. The activity of TyrR in *E. coli* is itself modulated by the presence and/or absence of aromatic amino acids [30,31,53]. The substrate for StlA is phenylalanine and, given the obligate requirement for TyrR in *stlA* expression, a simple model is that the presence of Phe, via TyrR, will positively affect *stlA* expression. However we did not see any affect of added Phe on the level of *stlA* expression in LB broth (data not shown). Unfortunately, *P. luminescens* TTO1 does not grow well in any defined minimal medium that we have tested and therefore we have not been able to test for any affect of Phe on *stlA* expression in the absence of high background levels of amino acids (Phe is present in LB broth at a concentration of approx. 1-2mM and the concentration does not change significantly during growth of *Photorhabdus* (our unpublished data)). The product of the StlA-catalysed reaction is CA and it has previously been reported that the presence of CA stimulates *stlA* expression i.e. there is a CA-dependent positive feedback loop [18]. In this study we have reported that the addition of 0.5mM CA to the culture medium significantly increases the expression of *stlA* after 72h (see Figure 4). It is not possible to speculate, at this time, whether CA is affecting *stlA* expression through an interaction with TyrR. However the *stlA-gfp* fusion is expressed normally even in the absence of CA (i.e. in the presence of the *stlA*::Tn10 mutation) suggesting that CA is not required for *stlA* expression (see Figure 4). Therefore, although not required for *stlA* expression, CA does appear to be involved in a positive feedback loop that increases *stlA* expression in *Photorhabdus*. 

**Figure 6:**
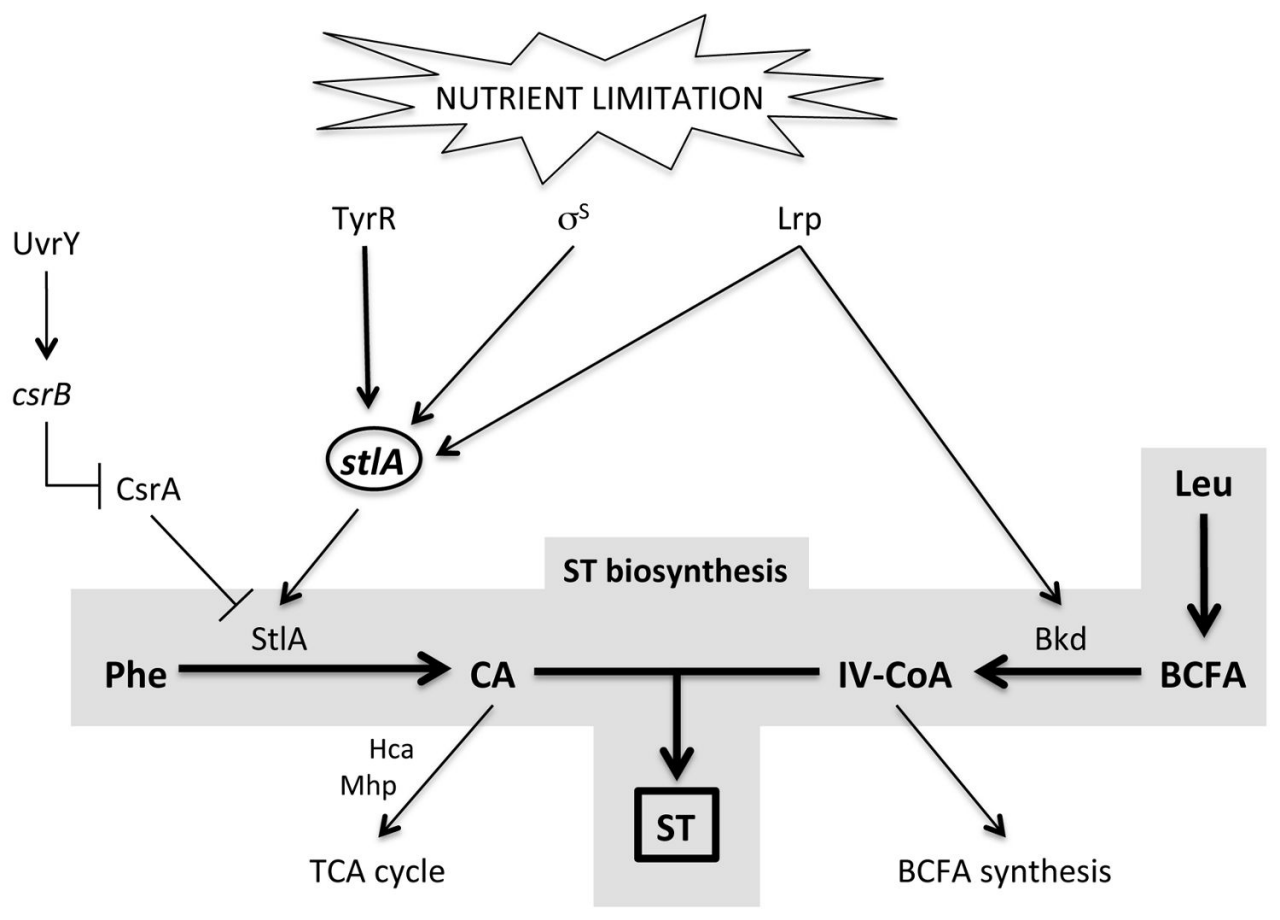
A proposed model for the regulation of *stlA* expression in *P. luminescens* TTO1. The expression of *stlA* is increased in response to nutrient limitation and we have identified 3 nutrient-associated regulators that all affect *stlA* expression. TyrR is required for *stlA* expression whilst σ^S^ and Lrp are needed for normal levels of *stlA* expression. Lrp also affects the levels of BCFA in the cell, presumably through a regulatory affect on *bkd* expression. The BarA/UvrY two-component pathway contributes to the post-transcriptional regulation of *stlA* expression, as described in the text. ST: 3’-5’-dihydroxy-4-isopropylstilbene; Phe: phenylalanine; CA: cinnamic acid; Leu: leucine; Bkd: branched-chain α-ketoacid dehydrogenase; BCFA: branched-chain fatty acids; IV-CoA: isovaleryl-CoA.

The *stlA* gene encodes the protein involved in the first step in ST biosynthesis and we have shown that *stlA* expression is controlled by a regulatory network that involves 3 transcriptional regulators i.e. TyrR, σ^S^ and Lrp (for our proposed model see Figure 6). We propose that these regulators link nutrient availability with *stlA* expression. Whilst TyrR is absolutely required for *stlA* expression (and ST production) both σ^S^ and Lrp appear to have a role in ensuring maximal levels of *stlA* expression under the appropriate environmental conditions. All of these regulators have been shown to play significant roles in the regulation of gene expression in response to the presence or absence of nutrients, in particular amino acids, in *E. coli* and other bacteria [29,31,45]. The important role for amino acids in the regulation of *stlA* expression is perhaps not surprising given that ST production requires both phenylalanine and leucine (the major regulatory co-factors of TyrR and Lrp, respectively, in *E. coli*). Indeed amino acid limitation, via the production of the alarmone ppGpp, has been shown to activate both Lrp and σ^S^ in *E. coli* and ppGpp also has an important regulatory role in the secondary metabolism of *P. fluorescens* and *Streptomyces coelicolor* A3(2) [40,54,55]. However, we have not yet established a role for ppGpp and the stringent response in the secondary metabolism of *Photorhabdus* and experiments are currently underway to address this. Nonetheless our work does provide an insight into the complex regulatory network that controls secondary metabolism, and therefore mutualism, in the increasingly important model organism, *Photorhabdus*. 

## Supporting Information

Figure S1 **Growth of TTO1 in LB broth can be divided into 4 distinct phases.** Wild-type TTO1 was cultured in LB broth in the wells of a 96-well microtitre plate and OD_600_ readings were taken at 15 min intervals and a growth curve was plotted. In this way 4 different growth phases (labeled I-IV) were readily identified. An indicative best-fit line for each phase was drawn by eye to aid in the visualization of the different phases.
(TIF)

Figure S2 **The production of ST is reduced in the Δ*lrp* mutant.** Wild-type and Δ*lrp* mutant was grown for 48h in LB broth and ST was isolated by organic extraction of the culture supernatant with ethyl acetate. Extracted samples were then separated using HPLC and the ST peak (indicated) was identified by detection with UV. The other peaks represent small metabolites present in the supernatants of TTO1 cultures, including CA and the anthraquinone pigment, AQ.
(TIF)

Figure S3 **There is an extended -10 box, associated with σ^S^-dependent promoters in *E. coli*, present in the *stlA* promoter in *P. luminescens* TTO1.** The *E. coli* consensus sequence for the extended -10 box is shown (taken from Weber et al. (2005); upper case represents strongly conserved positions and lower case represents less strongly conserved positions). In comparison is the sequence of the proposed extended -10 box in the *stlA* promoter (including the proposed -10 box (underlined)). Numbers indicate the nucleotide position relative to the proposed transcription start site.
(TIF)

Table S1 **Primers used for construction of deletion mutants.**
(DOCX)
