# Supplementary figures and images for: The Expression of stlA in Photorhabdus luminescens Is Controlled by Nutrient Limitation
Source: PLoS One. 2013 Nov 22;8(11):e82152. doi: 10.1371/journal.pone.0082152 (PMC3838401; doi:10.1371/journal.pone.0082152)

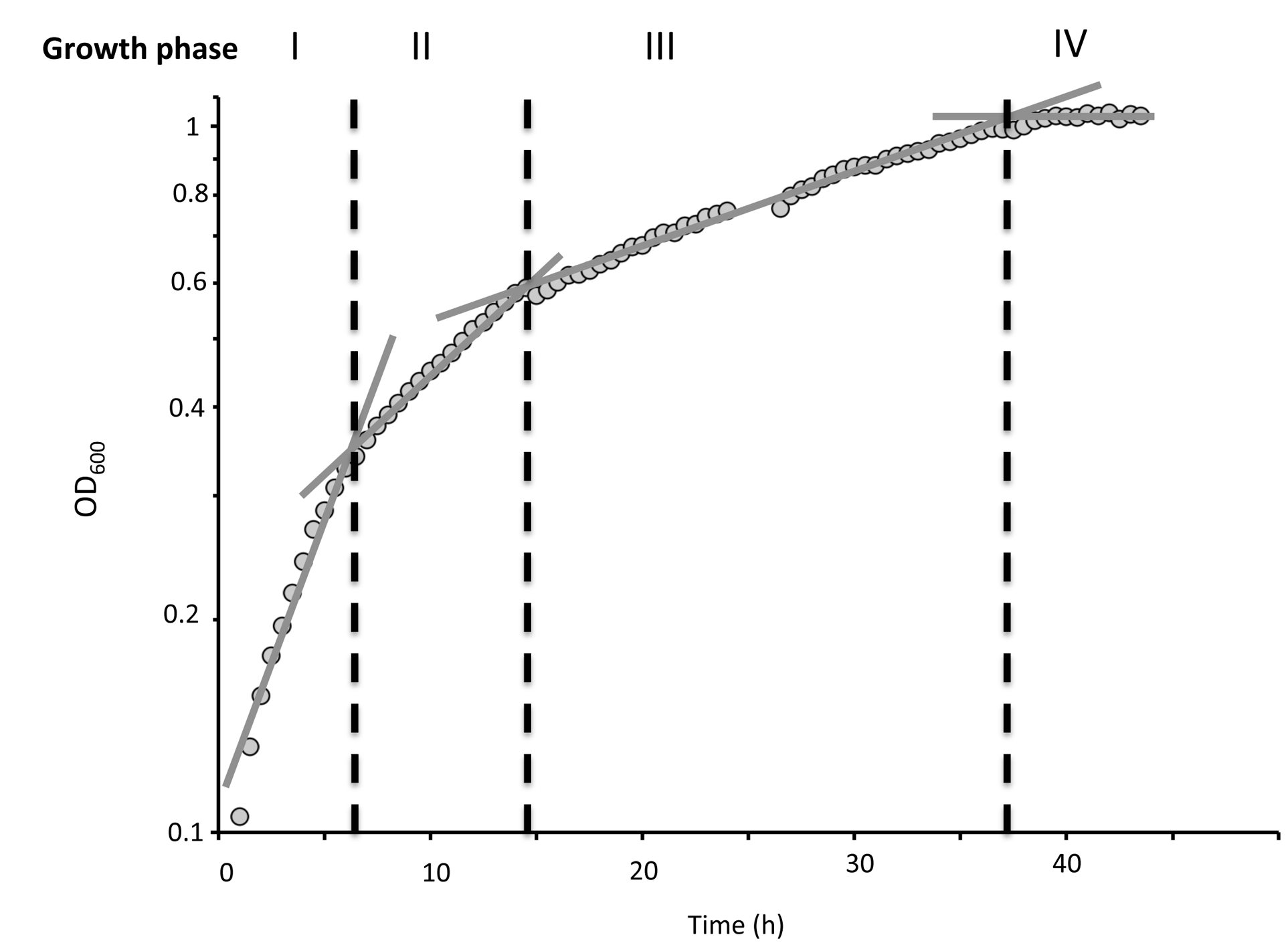

Supplement: Figure S1 — Growth of TTO1 in LB broth can be divided into 4 distinct phases. Wild-type TTO1 was cultured in LB broth in the wells of a 96-well microtitre plate and OD600 readings were taken at 15 min intervals and a growth curve was plotted. In this way 4 different growth phases (labeled I-IV) were readily identified. An indicative best-fit line for each phase was drawn by eye to aid in the visualization of the different phases. (TIF) [file pone.0082152.s001.tif]

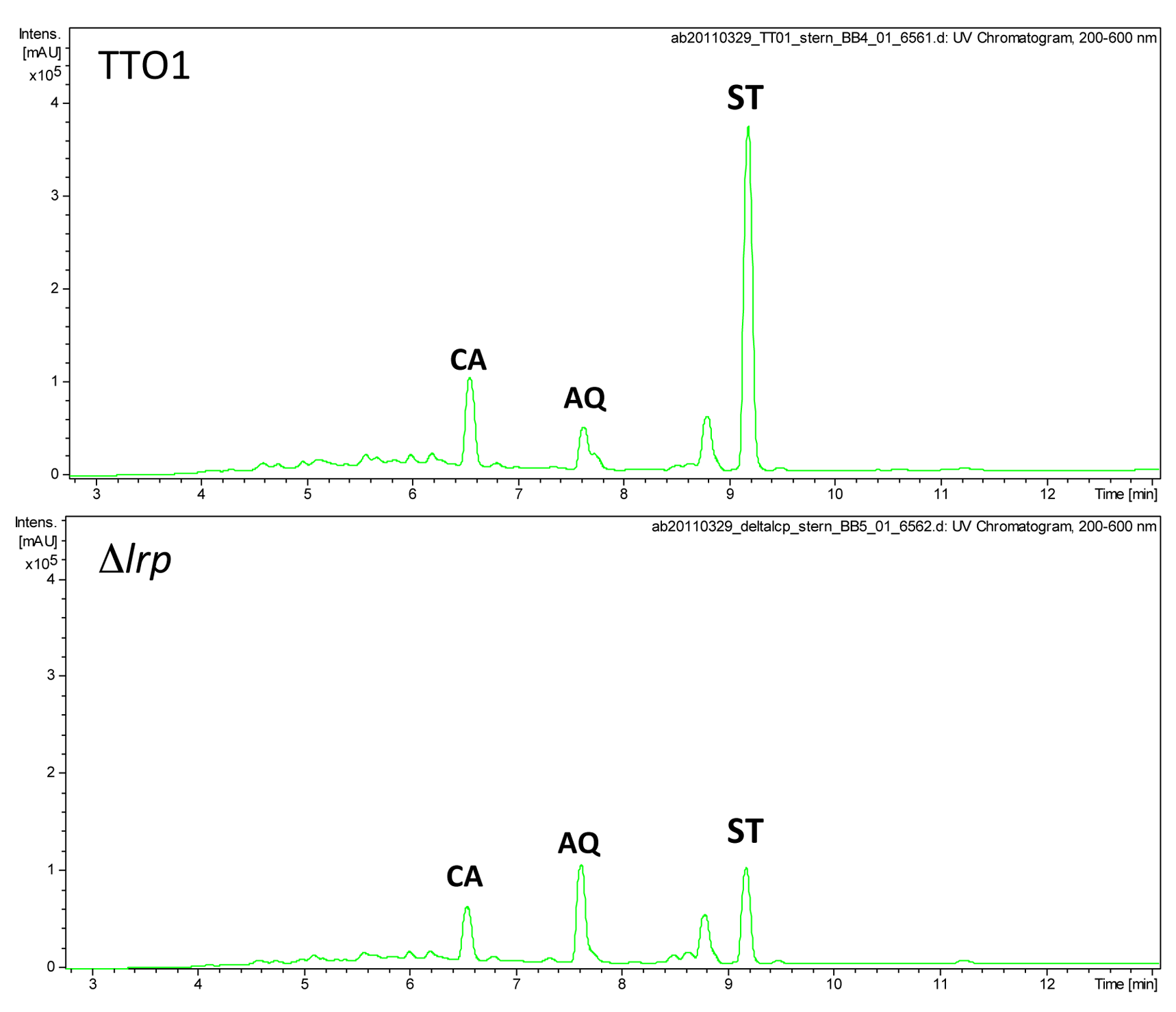

Supplement: Figure S2 — The production of ST is reduced in the Δlrp mutant. Wild-type and Δlrp mutant was grown for 48h in LB broth and ST was isolated by organic extraction of the culture supernatant with ethyl acetate. Extracted samples were then separated using HPLC and the ST peak (indicated) was identified by detection with UV. The other peaks represent small metabolites present in the supernatants of TTO1 cultures, including CA and the anthraquinone pigment, AQ. (TIF) [file pone.0082152.s002.tif]

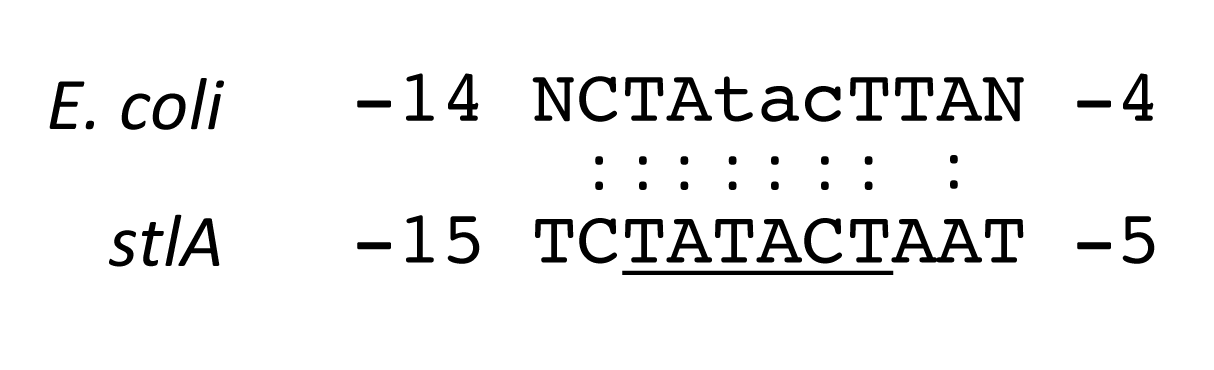

Supplement: Figure S3 — There is an extended -10 box, associated with σS-dependent promoters in E. coli, present in the stlA promoter in P. luminescens TTO1. The E. coli consensus sequence for the extended -10 box is shown (taken from Weber et al. (2005); upper case represents strongly conserved positions and lower case represents less strongly conserved positions). In comparison is the sequence of the proposed extended -10 box in the stlA promoter (including the proposed -10 box (underlined)). Numbers indicate the nucleotide position relative to the proposed transcription start site. (TIF) [file pone.0082152.s003.tif]
