# Supplementary material for: The Expression of stlA in Photorhabdus luminescens Is Controlled by Nutrient Limitation
Source: PLoS One. 2013 Nov 22;8(11):e82152. doi: 10.1371/journal.pone.0082152 (PMC3838401; doi:10.1371/journal.pone.0082152)
Supplement: Table S1 — Primers used for construction of deletion mutants. (DOCX) [file pone.0082152.s004.docx]

**Table S1**. Primers used for construction of deletion mutants.

| Primer | Sequence |
| --- | --- |
| barAA | 5’-ACC GCA TGC GAT ATC GAC GAA ATC CTA TCA CCA GTT GG-3’ |
| barAB | 5’-CTT AAT GTT CTA GAA TGT TCT ACA TAA TCG TCC ATT ATG G-3’ |
| barAC | 5’-CCA TAA TGG ACG ATT ATG TAG AAC ATT CTA GAA CAT TAA G-3’ |
| barAD | 5’-GTG GAG CTC CCG GGA ACG GTA ACG AAT TAC GTC GAA GC-3’ |
| uvrYA | 5’-ACC GCA TGC GAT ATC TAC AAC TCT CCC TGC ATG AGC TTG TCC-3’ |
| uvrYB | 5’-GTT TTT AAA AAT GTT TTT GAA TCA CAA AAA AGT GTC TCC AAC TGG C-3’ |
| uvrYC | 5’-GCC AGT TGG AGA CAC TTT TTT GTG ATT CAA AAA CAT TTT TAA AAA C-3’ |
| uvrYD | 5’-GTG GAG CTC CCG GGA GAC ACT ATC CTC ACA TTG TCG GAT AGG-3’ |
| tyrRA | 5’-TAA TAT CTG CAG GCC TGG CGT AAT ATC CGG CTA ATT AAT CG-3’ |
| tyrRB | 5’-TAA TATGAG CTC GCT GAG TGC CAG AAG GAA CTT CCG CAG C-3’ |
| tyrRC | 5’-GGT GGG CAT TGT TTT ACA TCT GTT TTG ACC TTA TTT ACA TTA ATA-3’ |
| tyrRD | 5’-GGT CAA AAC AGA TGT AAA ACA ATG CCC ACC GGA AGT GGG C-3’ |
| rpoSA | 5’-TAT TTA GCA TGC GAT TCT CAA TCA ACT AAT GC-3’ |
| rpoSB | 5’-GCC AAA CTA TTC AAC AAT TAC ATA AGC TGC CCC-3’ |
| rpoSC | 5’-GGG TAG GGG CAG CTT ATG TAA TTG TTG AAT AG-3’ |
| rpoSD | 5’-TAA ATA GAG CTC TTA TTG ACG CGT CGT TTA GG-3’ |
